# A Consultation Hospital

**Published:** 1900-02-17

**Authors:** 


### The Hospital

A Journal of

The Medical Sciences and Hospital Administration.

Vol. XXVII.—No. 699.] Edited by Sir Henry Burdett, K.C.B., and Solomon C. Smith, M.D., M.R.C.P. [February 17, 1900.

### A Consultation Hospital.

It is the boast of London charity that, in the presence of sickness, the difference between class and class is largely wiped away, and that by aid of the hospitals the poor as well as the rich have at their service all the resources of medical science. Yet, in the long list of medical charities there has been a gap, for no attempt seems to have been made until quite recently to provide for the poor those "consultations" and "second opinions" which form so important a factor in every-day practice among the rich. Charity has seemed to expend itself upon treatment, and the out-patient departments of the hospitals to be dominated by the ever-present necessity of prescribing bottles of physic for an untold number of trivial cases. We know, of course, that to any suggestion to open a purely consultative department the objection will be raised that consultations are luxuries, and that patients who are able to provide themselves with medicines, and probably with the attendance of a medical man, are not objects of charity, and are, therefore, outside the sphere of hospital work. It may be doubted, however, how far it is just to draw so distinct a line between the fit and the unfit, or to lay it down that a man who can pay a general practitioner has no excuse for coming to a charity when he wants the aid of a consultant.

No doubt in the beginning hospitals were established for the succour of the absolutely destitute. But that era is in this country long since past and gone. Since the establishment of the Poor Law medical service the continuance, the very existence even of the hospitals, is an admission that in sickness the poor cannot be divided by a strict line into paupers and payers, and that charity may properly give luxuries during illness to those who in health would not be fit objects of charity at all. The old definition, according to which a fit case for hospital treatment was one who was "unable to pay for medical attendance," has become to a large extent meaningless, seeing that anyone who is really unable to pay for medical attendance can have it supplied for nothing by the Poor Law authorities. The modern definition has come rather to be "a person who is unable to pay at the ordinary rate for the sort of help which his case requires," and according to this definition the patient whose case requires a consultation is just as much a fit "object of charity" as the one who requires an operation, or as the other to whom medicines are given with so liberal a hand. Few people who have not gone into this question have any idea how large a number of people there are who, while quite able to pay for ordinary medical attendance, either in the usual way or on some "provident" plan, are yet quite unable to pay the fees of the consultant. When this is recognised it becomes obvious that in going to hospitals in the hope of getting the opinion of some great man these people are not altogether and entirely "sponging on the charity," as some would have it. The hospital system probably forces them to carry away a bottle of physic; but what they want is the "opinion," and in trying to get it they are but doing in regard to consultations what is in practise generally admitted to be allowable in regard to surgical treatment—accepting as a charity what they cannot afford to obtain by payment. Unfortunately, while the patient who requires treatment finds all comfortably arranged for him, the one who asks only advice has to go through many troubles, and even then may fail to obtain the thing he seeks. He may no doubt get thoroughly examined, although not always by the great man he hoped to see, and no doubt also the bottle of physic given to him is a very good one; but that is not what he wanted.

It now seems as if this difficulty would be partly met, and this gap in medical charity would be to some extent filled up by a purely consultation hospital which has lately come into being under the name of the Polyclinic. Ostensibly, it is a medical college, having for its object the promotion of medical study among medical men after they have qualified for practice. It is well equipped, and, as the *Polyclinic*, the monthly journal of the institution, shows, it is already doing very good work. But it is something more than a college, and in its more recent development it is tending to become almost exactly the sort of consultation hospital, the lack of which forms, as we have said, a gap in the medical charities of London. The passport to its out-patient department is doubt as to the nature of the case, or rarity of the disease suffered from. Out-patient recommendation forms are now given to subscribers, as in the case of ordinary hospitals, but it seems to be the hope of the governing body that the majority of the patients attending will be sent up or brought up by their own medical men, and this will no doubt be the case when medical men discover that they can thus obtain for their poorer patients, and, be it added, for themselves, the same advantages in regard to a "second opinion" as their richer patients obtain by payment of fees. The only condition that is laid down seems to be that every case shall form the subject of a demonstration before the class of medical men attending the college. Perhaps it may be true that the primary object of giving these free consultations by eminent men is to get together a clinic of rare and interesting cases for teaching purposes. But by the very necessity of the case these consultations are becoming a large and important part of the work of the Polyclinic, and thus, by dint of its work meeting a well recognised but unfilled want, the institution is drifting into the position of a medical charity as well as a mere medical college, and while no doubt the teaching side will be well supported by the profession, it seems not unlikely that the charity side may assume such proportions as to make it appeal to charitable givers on the same basis as an ordinary hospital.

